# Nurse Leaders’ Perceptions of Existing Leadership Practices and Development Needs: A Descriptive Qualitative Study

**DOI:** 10.1155/jonm/1285019

**Published:** 2026-08-26

**Authors:** Estefanía Canedo, Carla Sílvia Fernandes, Paula Ferreira, María Manuela Martins

**Affiliations:** ^1^ Department of Nursing, Faculty of Nursing and Physiotherapy, University of Salamanca (USAL), Salamanca, Spain; ^2^ Nurse Department, School of Health, Polytechnic University of Viana do Castelo (UNIPVC), Viana do Castelo, Portugal; ^3^ NurseTechGames, Health Sciences Research Unit: Nursing (UICISA:E), Coimbra, Portugal; ^4^ School of Medicine and Biomedical Sciences, University of Porto (ICBAS-UP), Porto, Portugal, up.pt; ^5^ RISE-Health, School of Medicine and Biomedical Sciences, University of Porto, Porto, Portugal, up.pt

**Keywords:** education, leadership, management, nursing, professional development

## Abstract

**Aim:**

To explore nurse leaders’ perceptions of leadership practices and development needs.

**Background:**

Nursing leadership has gained increasing attention due to the growing complexity of healthcare systems and the need to ensure high‐quality patient care, staff retention and job satisfaction. However, recently many nurses assume leadership roles with limited preparation, highlighting the need to strengthen leadership development, education and training. Despite growing interest in nursing leadership, limited research has explored how nurse leaders perceive their roles, competencies and educational needs across different organisational contexts.

**Methods:**

An explorative, descriptive, qualitative design was adopted. Data were collected through face‐to‐face interviews from 28 nurse leaders working in hospital and primary care settings and analysed using reflexive thematic analysis following Braun and Clarke’s approach.

**Results:**

Four themes emerged: perceptions and meaning of the nurse leader role; leadership and management practices and competencies; leadership development pathways; and the role of leadership in care delivery and organisational processes. Across these themes, participants highlighted significant gaps in preparation for managerial and leadership responsibilities, particularly in areas such as communication, team motivation and staff development. These gaps were perceived as affecting job satisfaction, staff retention and the delivery of high‐quality patient‐centred care.

**Conclusions:**

The findings suggest that nurse leaders perceive leadership as a complex relational and organisational practice that extends beyond operational management. However, the predominance of experiential learning and perceived gaps in formal education indicate that current educational and organisational structures may not adequately prepare nurses for leadership responsibilities.

**Implications for Nursing Management:**

Nursing organisations and educational institutions should implement structured leadership development programmes, mentoring initiatives and competency‐based leadership training within undergraduate and continuing professional education to better prepare nurses for leadership responsibilities.

## 1. Introduction and Background

Administration, management and leadership are often perceived as referring to the same role and frequently used interchangeably in nursing literature and practice [1, 2]. However, they represent distinct yet interrelated dimensions that collectively shape the governance of healthcare systems and, more specifically, nursing care [1–3].

Nursing administration can be understood as a macro‐level framework for strategic institutional action [3], involving planning, organising and regulating operations to achieve organisational goals and anticipate potential problems [1, 2]. Nursing management is more commonly situated at the mezzo level [2, 3], relating to the tactical execution of these goals and encompassing decision‐making in financial, economic and political domains [3]. By contrast, leadership operates primarily at the micro level [4], emphasising interpersonal influence and the ability to motivate, inspire and guide individuals and teams towards shared objectives [2, 4].

The need to ensure job satisfaction, staff retention and high‐quality patient care has brought increasing attention to nursing leadership [5–9]. Beyond positional authority, leadership entails a commitment to patient well‐being, not only through the promotion of safe care practices but also through fostering the development of staff competencies [7, 10, 11]. These competencies extend across the diverse roles of nurses, from direct patient care to team management, education and research [12], highlighting their integral role in shaping contemporary nursing practice [11, 13, 14].

Effective leadership has consistently been associated with positive workforce and organisational outcomes, including motivation [15], increased job satisfaction, staff retention, team cohesion and quality of care. [16–19]. Conversely, poor leadership has been associated with professional dissatisfaction, turnover intentions [20–22] and poorer care outcomes [9]. This highlights the crucial role of nurse leaders in motivating their teams and fostering safe and supportive work environments [11, 12, 16, 23].

Moreover, the ethos of nursing leadership is grounded in caring relationships, not only with patients but also with staff [10, 24, 25]. When nurse leaders demonstrate authentic care and support, this contributes to improved team dynamics and better patient outcomes. Leadership should, therefore, be understood not only as a role but also as a relational and ethical practice guided by the values of dignity, integrity and mutual respect [10, 17].

At the same time, nurse leaders frequently experience tensions between their internal ethical responsibilities and external administrative pressures [7, 17, 26]. International health organisations such as the World Health Organisation (WHO) and the International Council of Nurses (ICN) have highlighted systemic challenges, including inadequate staffing, temporary contracts and poor compensation [22, 27]. These factors can contribute to work overload, exhaustion and occupational stress [28–30].

Recent studies have identified several factors that influence intrinsic motivation among nurses. Poor leadership and limited autonomy can contribute to burnout [16, 20, 21], while the absence of motivational factors, such as opportunities for lifelong learning, professional autonomy, responsibility and participation in decision‐making, may increase turnover intentions [6, 12]. Conversely, staff recognition and empowerment can foster retention, enhance workplace well‐being and improve the quality of care [16].

Within this study, leadership practices refer to the behaviours, responsibilities, competencies and strategies employed by nurse leaders in their daily managerial and leadership activities. Leadership development needs refer to the knowledge, skills, educational preparation and organisational support required to effectively fulfil these responsibilities and respond to contemporary healthcare challenges.

The Spanish public healthcare system provides universal access and has increased its nursing workforce by 22.7% between 2014 and 2022 [31, 32]. However, it operates under new public management models that prioritise efficiency [30]. This shift has contributed to an increase in temporary contracts, reaching 80.7% in 2023 and mainly affecting newly registered nurses. These precarious employment conditions negatively affect job satisfaction [33] and may ultimately compromise care quality. Consequently, a sufficient and stable nursing workforce is essential to ensure high‐quality care, and supportive workplace relationships are crucial for creating positive work environments and a strong culture of care [10, 34].

In this context, nurse leaders occupy a strategic position between organisational demands, workforce management and the delivery of high‐quality care. Understanding their perceptions is, therefore, particularly relevant for identifying current leadership challenges within the Spanish healthcare system.

At the same time, the global nursing shortage has intensified these challenges, disrupting care delivery, reducing the quality of nursing care and negatively affecting patient satisfaction and treatment outcomes [22, 34]. Nurse leaders play a critical role in maintaining the sustainability of healthcare systems [11, 22]. By fostering resilience both in themselves and within their teams, they contribute to nurse retention and organisational stability [14].

Leadership development has, therefore, become a central priority in nursing [10, 11]. Structured leadership training has been associated with improved communication, decision‐making, tconflict management and team coordination [8, 10, 12, 17], enabling nurse leaders to perform their managerial and organisational roles more effectively [25, 26]. Conversely, insufficient preparation for leadership roles may contribute to role strain, ineffective team management, increased turnover intentions and poorer organisational outcomes [6, 9, 15]. For this reason, several authors advocate the progressive development of leadership competencies through both undergraduate education and continuing professional development [11, 13, 20, 22, 35].

Although the literature has extensively examined leadership styles, outcomes and education, considerably less attention has been paid to how nurse leaders themselves perceive their day‐to‐day leadership practices and development needs. Understanding these perceptions is essential for informing leadership education, professional development initiatives and organisational strategies aimed at strengthening nursing leadership.

## 2. Aim

The study explored nurse leaders’ perceptions of existing leadership practices and development needs.

## 3. Methods and Materials

### 3.1. Study Design

An exploratory descriptive qualitative design was used to understand how nurse managers and leaders perceive their roles, leadership practices and development needs. This methodological approach is particularly suitable for exploring experiences, meanings and perceptions that cannot be adequately captured through quantitative methods. It provides an in‐depth understanding of complex professional and organisational phenomena within specific contexts [36]. Rigour was ensured through adherence to the Consolidated Criteria for Reporting Qualitative Research (COREQ) guidelines [37].

### 3.2. Contexts

This study was conducted in the largest of the 17 Spanish regions. Two healthcare settings were included: a university hospital complex and a primary healthcare network. Within the primary healthcare network, most institutions provide services to rural populations. These settings were purposively selected to capture diverse organisational contexts within the public healthcare system and to reflect the diversity of structures in which nurse leaders perform their roles and responsibilities.

The inclusion of different settings enabled the exploration of a range of organisational conditions and leadership experiences. This diversity was considered relevant for examining nurse leaders’ perceptions of their roles, responsibilities and professional preparation across different healthcare structures.

### 3.3. Participants and Sampling

Participants were recruited between December 2022 and February 2023. Purposive sampling was initially used to recruit information‐rich participants with direct experience of leadership and management relevant to the study aim. Snowball sampling was subsequently employed to identify additional nurse leaders with similar professional experience and facilitate access to additional participants who could provide in‐depth insights into the phenomenon under investigation [38]. Initial invitations were sent by email through the researchers’ professional networks. Individuals who expressed interest contacted the research team to confirm their availability for an on‐site interview. Upon agreeing to participate, they received detailed information regarding the study objectives and procedures.

The study included nurse leaders working at different organisational levels within the participating institutions. These roles comprised supervisors, who work in close contact with clinical teams without formal managerial responsibilities; team managers, who are responsible for the management of specific care units; and unit managers, who oversee broader clinical areas. For consistency throughout the manuscript, the term ‘nurse leaders’ was used as an umbrella concept to refer to all leadership positions across organisational levels.

Interviews were scheduled only after participants had confirmed their willingness to take part. All interviews were conducted by a nursing student who had no prior professional relationship with the participants and were supervised by a member of the research team. The inclusion criteria were as follows: (I) being a nurse leader currently occupying a leadership position within the participating institution and (II) having at least one year of experience in a leadership or management role.

Nurses who did not hold leadership responsibilities or who had less than one year of experience in a leadership role were excluded to ensure that participants had sufficient experience to reflect on leadership practices and responsibilities.

### 3.4. Data Collection and Management

Face‐to‐face interviews were conducted using a semistructured interview guide by a nursing student under the supervision of a research team member. Prior to each interview, participants received information about the study and gave their informed consent. All interviews were audio‐recorded, conducted in Spanish, lasted between 45 and 60 min and took place at the nurse leaders’ workplaces during working hours.

At the beginning of each interview, the nursing student provided a brief explanation of the study and allowed time for questions. Subsequently, consent for audio recording was confirmed before each session. Field notes were taken during the interviews to capture nonverbal cues, contextual observations and relevant comments.

No pilot interviews were conducted. However, consistent with the study design, the semi‐structured interview guide was developed based on the research questions and theoretical framework [36]. Probing techniques were used throughout the interviews to elicit further detail and clarification, including follow‐up questions and prompts for elaboration, in line with qualitative interviewing practices aimed at enhancing the depth and clarity of the data [36].

At the end of each interview, participants were given the opportunity to add any additional information they considered relevant. To ensure researchers’ reflexivity, continuous critical reflection was undertaken on its potential influence on data collection and interpretation, including awareness of prior assumptions and professional backgrounds. Field notes supported this process, and regular discussions within the research team supported reflexive examination of emerging findings, thereby enhancing credibility and transparency [36, 39].

Data saturation was reached after 22 interviews, as no new themes or relevant information emerged. Nevertheless, all scheduled interviews were conducted to confirm the consistency and robustness of the findings, in line with qualitative research standards [39–41]. Finally, 28 participants completed the interviews.

All interviews were transcribed verbatim into text manually by the nursing student who conducted the interview within 24 h. The research team reviewed the audio recordings and cross‐checked the transcripts to ensure accuracy and completeness. The data were then imported into Atlas.TI software to support data management, organisation and coding. Quotations included in the manuscript were translated into English by the research team and cross‐checked to ensure conceptual and semantic equivalence.

### 3.5. Data Analysis

Data were analysed using Braun & Clarke reflexive thematic analysis, following the proposed six‐phase approach [39–41]. This method was selected due to its flexibility and suitability for exploring patterns of meaning within qualitative data.

The first phase involved familiarisation with the data (I), reading and rereading the data, note‐taking and writing emerging ideas. In the second phase, coding (II), involved marking and labelling fragments of the textual information that held any meaning or answered the research questions [39–41]. Given the study’s theoretical framework and analytical focus on nursing management and leadership, a hybrid coding approach combining deductive and inductive strategies was adopted [42].

Initially, deductive coding was conducted, generating 98 codes based on predefined concepts derived from the theoretical framework, including leadership competencies, communication, motivation, human resources management, organisational management, quality of care and leadership development. This process was informed by a prior documentary analysis, which enabled the identification of initial codes, relationships and meanings within the literature [43]. Subsequently, an inductive approach was used to analyse previously uncoded data segments, allowing for the identification of new codes and the emergence of additional patterns of meaning [41].

In the third phase, codes were grouped into initial themes (III) based on conceptual similarity and relevance to the research questions. At the next step, themes were reviewed and refined (IV) to ensure coherence within themes and consistency across the dataset, resulting in the development of a thematic map. In the fifth phase, themes were clearly defined and named (V), capturing their core meanings and scope. Finally, the sixth phase involved producing the report (VI), integrating the analytical findings with the research questions and supporting them with representative data extracts [39–41].

Braun and Clarke’s [39–41] proposal provided a consistent process for this study. Combining deductive and inductive approaches enabled a comprehensive and nuanced analysis, integrating existing theoretical perspectives with insights emerging directly from the data [39–41].

### 3.6. Trustworthiness and Rigour

Trustworthiness was ensured following the credibility, dependability, confirmability and transferability criteria proposed by Lincoln and Guba [42–46]. Credibility was enhanced through face‐to‐face semistructured interviews, the use of field notes to capture contextual observations, prolonged engagement with the data through repeated reading of the transcripts and regular reflexive discussions within the research team during data collection and analysis. Dependability and confirmability were supported by applying Braun and Clarke’s [39, 41] reflexive thematic analysis through a transparent six‐phase analytical process.

Data were initially coded by one researcher and subsequently discussed with the research team to refine codes, develop themes and enhance analytical consistency. Atlas.ti was used to support systematic data management and organisation throughout the analytical process. Transferability was strengthened by providing a detailed description of the study context, participants, sampling strategy and analytical procedures, enabling readers to determine the applicability of the findings to similar healthcare settings. Methodological transparency was further enhanced through adherence to the COREQ guidelines [37].

### 3.7. Ethical Considerations

The ethical committees of the Pontifical University of Salamanca (CEI‐24.11.2022) and the University Health Care Complex of Salamanca (CAUSA‐19.12.2022) approved the research protocol. All procedures were conducted in accordance with the principles of the Declaration of Helsinki, ensuring voluntary participation, informed consent, confidentiality and anonymity of participants and the right to withdraw from the study at any time without consequences. Before the interviews, all participants were provided with written and oral information and were allowed to choose their time and place. Informed consent was obtained from each participant, and all participants received a code (H1–18 and P1–10) when transcribing interviews.

Interview recordings and transcripts were stored on password‐protected institutional devices accessible only to the research team. All data were anonymised prior to analysis and handled in accordance with applicable data protection regulations, including the General Data Protection Regulation (GDPR). Personal identifiers were removed during transcription, and all study data will be retained for 10 years in accordance with institutional data management policies, after which they will be securely destroyed.

Some participants had a prior professional relationship with members of the research team, as a small number also held positions as associated lecturers. However, no hierarchical relationship existed between researchers and participants at the time of the study.

## 4. Results

### 4.1. Participants’ Demographic Characteristics

A total of 28 participants were included; the majority were female (*n* = 26). The mean age was 48.7 years (SD = 7.8), with a mean total nursing experience of 25.6 years (SD = 6.5) and a mean leadership experience of 4.5 years (SD = 4.2). Most participants held middle management positions (team managers), followed by supervisors, who did not hold formal leadership positions and a small number of unit managers.

### 4.2. Thematic Analysis

The analysis resulted in four themes and eight subthemes, supported by a comprehensive coding framework derived through a hybrid deductive–inductive process. These themes represent shared patterns of meaning related to nurse leaders’ perceptions, competencies, development pathways and managerial responsibilities. Although participants occupied different leadership positions (supervisors, team managers and unit managers), the analysis focused on identifying shared perceptions across these roles rather than comparing organisational levels. Therefore, the term ‘*nurse leaders’* was adopted as an umbrella concept, encompassing all leadership positions included in the study. A summary of the themes and subthemes is presented in Table 1.

**TABLE 1:** Summary of themes and subthemes.

Theme	Subthemes
1. Perceptions and meaning of the nurse leader role	1.1 Thoughts on nurse management and leadership 1.2 Meaning of being a nurse manager/leader

2. Leadership and management practice and competencies	2.1 Clinical management and leadership 2.2 Being a leader 2.3 Leadership and management of human resources

3. Leadership development pathways	3.1 Experiential learning 3.2 Gaps in formal education

4. Role in care delivery and organisational processes	4.1 Logistics and stock control management 4.2 Quality of care improvement 4.3 Leading the change

#### 4.2.1. Theme 1: Perceptions and Meaning of the Nurse Leader Role

This theme captures how participants conceptualise the role of nurse leadership and management, emphasising its importance and associated responsibilities within healthcare organisations. Ethical and professional values were consistently highlighted, particularly in relation to promoting team well‐being and ensuring a supportive work environment. Two subthemes emerged under this theme, as follows: (I) Thoughts on nurse management and leadership; and (II) Meaning of being a nurse manager/leader.

##### 4.2.1.1. Subtheme 1: Thoughts on Nurse Management and Leadership

Participants consistently described their role as essential for organisational functioning, quality of care and team coordination, although it was often perceived as undervalued.

 7P: Necessary job position in which we nurses are always deeply committed and do so much to improve services.
 1P: Important role that solves a lot of problems, and should be more recognised, maybe even by conducting a study to highlight its value.

##### 4.2.1.2. Subtheme 2: Meaning of Being a Nurse Manager/Leader

The role was framed as a responsibility requiring accountability, fairness and a global organisational perspective. Participants emphasised the importance of fostering a team‐oriented culture, recognising team members as partners and linking team performance with patient outcomes.

 9P: A responsibility to ensure that everything works well at the team level, at the operational level, in terms of day‐to‐day and at the overall organisation.
 11H: A personal challenge; it involves improving the work of my team, and this is what I focus on every day: doing a good job, making sure everything runs smoothly and trying to ensure everyone feels comfortable.

#### 4.2.2. Theme 2: Leadership and Management Practice and Competencies

This theme captures the leadership practices and competencies that were perceived as essential for the effective management of nursing teams and care processes. Participants emphasised that their leadership knowledge and skills were central to shaping the work environment, noting that individual personality and temperament influence how decisions are approached, whether through consensus, negotiation or directive action. Three subthemes emerged from this theme: (I) Clinical management and leadership; (II) Being a leader; and (III) Leadership and management of human resources.

##### 4.2.2.1. Subtheme 1: Clinical Management and Leadership

Participants emphasised the importance of structured processes, anticipation and prioritisation to ensure efficient and safe care delivery.

 12H: Procedures must be very, very clear, concise and well‐defined; otherwise, conflicts with staff will arise.
 1P: Structured, organised, documented; everything is written down and out in open view, anticipation.

##### 4.2.2.2. Subtheme 2: Being a Leader

Leadership was associated with interpersonal competencies, particularly communication, active listening and conflict management, as well as the ability to support and guide teams. Establishing clear boundaries was also identified as essential for maintaining a structured and effective working environment.

 16 H: A leader must be a friendly person who sets limits and establishes rules.
 10P: Communication above all else. I like things to run smoothly, and I try to be as fair as possible when it comes to what needs to be done and what my nursing team has to take on.

##### 4.2.2.3. Subtheme 3: Leadership and Management of Human Resources

Managing human resources was described as one of the most complex aspects of the role, involving staff allocation, integration and retention, as well as fostering a positive work environment. Participants highlighted that team cohesion and well‐organised procedures contribute significantly to effective teamwork and the delivery of high‐quality care.

 18H: If I am not at ease in my job, I do not perform well enough, I do not provide adequate patient care, I am not a good colleague; but when you arrive at work, and your personal life is somewhat sorted out and your needs are met, then you work much better.
 1P: I really enjoy working on that, making it clear that today I need your help, or tomorrow you might need mine or sharing concerns about work or patients. I think it is so important to work as a team.

#### 4.2.3. Theme 3: Leadership Development Pathways

This theme highlights participants’ perceived leadership development needs, including educational preparation, experiential learning and ongoing professional support. Participants consistently underscored the need to prioritise structured leadership education within undergraduate nursing curricula, recognising leadership as a core component of professional nursing practice. Two subthemes emerged from this theme: (I) Experiential learning; and (II) Gaps in formal education.

##### 4.2.3.1. Subtheme 1: Experiential Learning

Participants frequently described leadership competencies as being acquired primarily through experience, often describing themselves as ‘self‐taught’ or ‘autodidactic’. This reflects a reliance on informal learning processes to develop the competencies required for leadership roles.

 7P: I have learnt from experience, not from nursing education.
 16H: I started through trial and error, taking courses on my own, identifying my weaknesses and needs; essentially, I taught myself.

##### 4.2.3.2. Subtheme 2: Gaps in Formal Education

A lack of structured leadership training during undergraduate education and limited access to professional development opportunities were consistently reported. While not all nurses assume formal leadership positions in their lifetime, participants emphasised that competencies related to resource management and organisation of care are inherent to nursing practice and should, therefore, be addressed during undergraduate training.

 9H: We are not all natural leaders; in my view, I arrived here with very limited training. We are not well trained for this in university.
 13H: The undergraduate level is the time to do it.

#### 4.2.4. Theme 4: Role in Care Delivery and Organisational Processes

This theme captures the operational and organisational responsibilities associated with the nurse leader role in ensuring effective care delivery. Participants also emphasised the importance of grounding management practices in scientific evidence, including research and needs assessment, as well as understanding the rationale behind resource utilisation and selecting the most effective and efficient interventions. Achieving a balance between costs and clinical benefits was considered essential for ensuring high‐quality care and responsible resource management. Three subthemes were derived from this theme: (I) Logistics and stock control management; (II) Quality of care improvement; and (III) Leading the change.

##### 4.2.4.1. Subtheme 1: Logistics and Stock Control Management

Participants highlighted the complexity of managing material resources, emphasising the need for efficiency, anticipation and responsible use to avoid disruptions in care. This responsibility was described as increasingly challenging within the current healthcare context.

 12H: If you run out of one material, you stop a whole chain of work, and in the end, it is the patient who suffers the consequences.
 13H: You have to keep a very close eye on things because you always need to be on top of your spending, but there are certain things you cannot do without—it would be chaotic—so you have to stay on top of it.

##### 4.2.4.2. Subtheme 2: Quality of Care Improvement

Participants consistently highlighted their critical role in ensuring the effective functioning of healthcare services. Leadership was strongly associated with improving care quality through supervision, adherence to protocols and promotion of evidence‐based practice. These practices were perceived to foster trust and confidence, contributing to a supportive work environment and improved care outcomes.

 3P: Looking closely at the protocols, keeping good records and handling programmes correctly, to carry out specific care, providing a service of high quality.
 10P: Through ongoing training and joint clinical sessions. Ensuring that when there is something new that needs to be introduced, the whole team and nursing staff are informed, and that everyone has access to this information.

##### 4.2.4.3. Subtheme 3: Leading the Change

Participants described change as an inherent component of leadership, requiring adaptability, clear communication and team involvement in decision‐making. Change was not perceived as a disruption but rather as an ongoing process of optimisation and adjustment within the organisational system.

 11H: Changes are easier when the team is involved.
 13H: The final decision is mine, but we always reach a consensus, and if anything needs changing, I have a system in place called ‘suggestions’: if anyone wants to propose something, they just write it down—no problem—we have very open communication.

Overall, the findings highlight the multifaceted nature of the nurse leader role, encompassing interpersonal, organisational and developmental dimensions. Across themes, leadership and management practices were closely linked to team coordination, work environment optimisation and quality of care improvement, while revealing persistent gaps in formal preparation and a reliance on experiential learning. These interconnected dimensions illustrate the complexity of nursing leadership within contemporary healthcare settings.

## 5. Discussion

The findings suggest that nurse leaders perceive leadership as a multifaceted role that extends beyond operational management and encompasses ethical, interpersonal, organisational and developmental dimensions. Rather than viewing leadership and management as separate domains, participants perceived them as interconnected dimensions of professional nursing practice that directly influence workforce well‐being and quality of care. These findings are consistent with the recent literature emphasising leadership as a relational and value‐driven practice that directly influences care quality and team performance [10, 17].

Despite this positive perception, participants also described the role as demanding and challenging, particularly in contexts characterised by excessive workload and limited recognition. Similar findings have been reported in previous studies, where a lack of recognition and organisational support negatively affected motivation and contributed to feelings of professional isolation [17, 30, 44].

Participants associated effective leadership with interpersonal competencies, particularly communication, conflict management and team coordination. Participants highlighted the importance of fostering collaborative and interdisciplinary work, reinforcing evidence that positive interpersonal relationships are central to creating supportive work environments and enhancing staff well‐being [8, 16, 17].

Autonomy emerged as a critical factor for both leaders and teams. However, consistent with existing literature, autonomy alone was insufficient to sustain effective practice without adequate support, information and training [11, 17, 22, 47]. This finding underscores the need for balanced leadership approaches that integrate empowerment with structured guidance.

Human resource management was identified as one of the most complex aspects of leadership, particularly regarding staff motivation, retention and workload distribution. These findings align with previous research highlighting the central role of nurse leaders in shaping work environments and reducing turnover intentions [6, 12, 34].

The findings support the ongoing debate regarding whether leadership is innate or developed, with participants clearly emphasising the importance of formal education and training. In line with previous studies, participants reported insufficient preparation during undergraduate education and limited access to continuing professional development opportunities [2, 48, 49].

Leadership competencies, such as communication, problem‐solving and organisational skills, are widely recognised in the literature as essential for effective practice [2, 11, 12, 50]. However, their development remains inconsistent across educational programmes [51]. Participants’ reliance on experiential learning reflects a gap between academic preparation and practice demands, highlighting the need to integrate leadership development more systematically into nursing curricula.

Lifelong learning was also identified as critical, particularly within evolving healthcare systems [51]. Evidence suggests that structured leadership programmes and mentoring initiatives have been shown to enhance leadership capacity, improve decision‐making and support the management of complex organisational and ethical challenges [17, 47].

Participants emphasised the complexity of operational responsibilities, including resource management, quality improvement and leading organisational change. While the management of material resources was recognised as demanding, it was not perceived as the greatest challenge, consistent with previous findings [35, 47].

The quality of care emerged as a central concern, influenced by leadership practices, team dynamics [52] and organisational conditions. Communication, empathy and team cohesion were identified as key drivers of care quality, supporting evidence linking leadership behaviours to patient outcomes and workplace environments [12, 16, 21].

Teamwork was repeatedly highlighted as fundamental, with participants emphasising that effective care delivery depends on collective engagement. This aligns with the literature, associating team cohesion with transformational and motivational leadership approaches [8, 11, 16, 52].

Moreover, leading change was recognised as an inherent aspect of leadership, requiring adaptability, effective communication and team involvement. Consistent with previous research, participants emphasised that inclusive decision‐making facilitates acceptance and implementation of change, strengthening team engagement and organisational outcomes [17].

Overall, the findings highlight the complexity of the nurse leader role, which extends beyond operational management to include relational, ethical and developmental dimensions. A notable contribution of this study is the identification of nursing leadership as an integrated practice in which team management, quality improvement, professional development and organisational responsibilities are perceived as closely interconnected rather than distinct functions.

These findings demonstrate that leadership and management influence team functioning, work environment quality and care outcomes, while revealing persistent gaps in formal preparation and a continued reliance on experiential learning. Collectively, these results reinforce the need for integrated leadership development strategies that combine formal education, organisational support and experiential learning opportunities to strengthen nursing leadership in contemporary healthcare systems.

## 6. Strengths and Limitations

This study has several strengths. Adherence to COREQ ensured methodological transparency and rigour. Data were collected through face‐to‐face interviews, which allowed for an in‐depth exploration of participants’ experiences. Additionally, the use of audio recordings and field notes enhanced the completeness of the data and provided a better contextual understanding. The involvement of multiple researchers in data collection and analysis further strengthened reflexivity and reduced the risk of individual bias.

Nevertheless, some limitations should be acknowledged. As a qualitative study, the findings are context‐specific and not intended to be generalisable, but rather transferable to similar settings. The use of interviews as the sole method of data collection may be subject to self‐report bias, including social desirability and selective recall. Additionally, participants’ responses may have been influenced by their professional roles or organisational context. Although efforts were made to ensure openness and confidentiality, the presence of the researcher during interviews may have influenced participants’ willingness to disclose certain perspectives. Similarly, some prior professional relationships may have influenced participants’ responses despite the measures adopted to minimise this risk.

Furthermore, all participants were recruited from a single region in Spain, which may limit the transferability of the findings to other healthcare systems or cultural contexts. The use of purposive and snowball sampling may also have introduced selection bias, as participants with a particular interest in leadership and management may have been more likely to participate. Additionally, the predominance of middle management participants may have restricted the representation of perspectives from higher organisational levels. Future research should consider including a broader range of leadership positions and healthcare settings.

## 7. Conclusions

This study provides insights into how nurse leaders perceive their leadership practices and development needs within contemporary healthcare settings. The findings suggest that leadership is perceived as an integrated practice that combines relational, organisational, ethical and quality‐improvement responsibilities rather than a purely managerial function.

Participants identified effective communication, team engagement, supervision and organisational coordination as central leadership practices while highlighting persistent development needs related to leadership preparation, ongoing professional development, mentorship and organisational support. These findings reinforce the importance of strengthening leadership development across both undergraduate education and professional practice.

By highlighting nurse leaders’ perspectives, this study contributes to a deeper understanding of the competencies and support structures required to sustain effective nursing leadership and promote high‐quality care in increasingly complex healthcare systems. Future research should explore these perceptions across different organisational levels, including hospital administrators and executive board members.

## 8. Implications for Nursing Management and Leadership

This study highlights several implications for nursing management, leadership practice and leadership development.

### 8.1. Leadership Development and Education

⁃ Leadership and management competencies should be systematically integrated into undergraduate nursing education and continuing professional development.
⁃ Leadership development should combine formal education, mentorship, experiential learning and organisational support.

### 8.2. Leadership Practice and Organisational Support

⁃ Nurse leaders should foster healthy work environments through effective communication, shared decision‐making and team involvement.
⁃ Team empowerment and professional development should be prioritised to sustain motivation, retention and quality of care.
⁃ Resource management should be recognised as a shared professional responsibility, supported by appropriate training and technological innovation.

## Author Contributions

Estefanía Canedo: study design; conceptualisation and investigation; data collection, curation and formal analysis; writing–original draft; writing–review and editing. Carla Sílvia Fernandes: conceptualisation; data curation; supervision; writing–review and editing. Paula Ferreira: methodology and formal analysis; supervision; data curation; validation; writing–review and editing. María Manuela Martins: study design; methodology; conceptualisation and investigation; data curation and formal analysis; validation; writing–review and editing.

## Funding

No funding was received for this research.

## Conflicts of Interest

The authors declare no conflicts of interest.

## Data Availability

Data supporting the study findings are available from the corresponding author upon reasonable request. The data are not publicly available due to the privacy of participants and ethical restrictions.
